# Wrist arthro-CT: don’t forget to check the foveal attachment

**DOI:** 10.1186/s13244-026-02327-z

**Published:** 2026-06-24

**Authors:** Julien Dejean-Servieres, Stéphanie Delclaux, Elorie Adamski, Hélène Chiavassa-Gandois, Franck Lapegue, François Lafourcade, Céline Goumarre, Antoine Filliole, Constance Lambeaux, Viet-Tam Van, Rokia El Khalfi, Léo Millet, Elise Bouko-Levy, Nicolas Sans, Marie Faruch-Bilfeld

**Affiliations:** 1 Service de Radiologie, CHU Toulouse-Purpan, Toulouse, France; 2 Département d’orthopédie et traumatologie, CHU Toulouse-Purpan, Toulouse, France; 3 Service de gynécologie médicale, CHU Toulouse-Purpan, Toulouse, France

**Keywords:** Wrist injuries, Triangular fibrocartilage complex, Tomography (X-ray computed), Diagnostic errors, Foveal injuries

## Abstract

**Objective:**

The aim of this study was to demonstrate that foveal lesions of the triangular fibrocartilage complex (TFCC) are underreported on wrist arthro-CT and to compare imaging review data with clinical and surgical findings to assess their impact on patient management.

**Materials and methods:**

One hundred forty-nine wrist arthro-CTs were reviewed by two musculoskeletal radiologists, who were blinded to the surgical data and initial reports. Discrepancies between TFCC lesions and the initial reports were analysed and compared with the clinical and surgical data.

**Results:**

TFCC lesions were identified in 59% of patients, including 43% ulnar, 33% central, and 3% radial lesions. A comparison with the initial reports revealed 45 underreported lesions, 89% of which involved ulnar lesions, including 69% affecting the foveal bundle. Patients with complete ulnar TFCC lesions reported significantly more ulnar-sided pain and radioulnar instability than those without TFCC lesions (*p* < 0.0001). Isolated foveal lesions were significantly associated with ulnar-sided pain (*p* = 0.0201), but not with radioulnar instability (*p* > 0.99). Among the 149 patients, 12 underwent TFCC surgery. In 8 patients, the lesions were identified in the initial radiological reports and confirmed intraoperatively. In 4 patients, surgery was performed despite negative initial reports. A retrospective imaging review confirmed the lesions, which were in agreement with the surgical findings.

**Conclusion:**

The application of new classifications and recent knowledge enabled the identification of previously unreported lesions in 28% of cases, including 69% involving the foveal attachment, highlighting the diagnostic challenges of this type of injury.

**Critical relevance statement:**

This study shows that foveal TFCC lesions are frequently underreported on wrist arthro-CT and that a structured review, incorporating recent classifications and multiplanar reconstructions, significantly improves lesion detection, particularly for foveal involvement.

**Key Points:**

- Foveal TFCC lesions are difficult to identify on wrist arthro-CT because of their subtle and variable imaging appearance.
- Re-evaluation of arthro-CT scans and comparison with initial reports, which used recent classifications and multiplanar reconstructions, revealed that 28% of the lesions were underreported.
- Improved detection of foveal involvement may lead to more accurate management of ulnar-sided wrist pain.

**Graphical Abstract:**

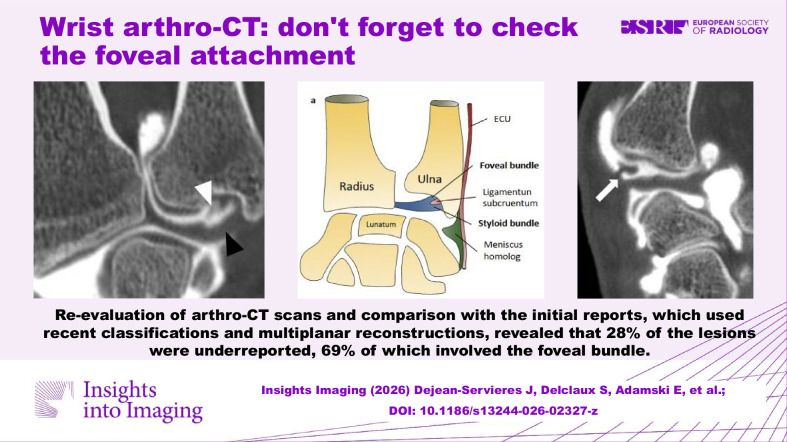

## Introduction

Traumatic wrist conditions are common, and the detection of lesions, as well as the assessment of their clinical relevance, remains a diagnostic challenge for clinicians [1]. Ulnar-sided wrist pain has multiple causes but is most frequently associated with injuries of the triangular fibrocartilage complex (TFCC) [2]. A detailed understanding of the radiological anatomy and the spectrum of lesions detectable on imaging in this region is essential for optimal patient management.

### Anatomical and clinical picture

**Fig. 1:**
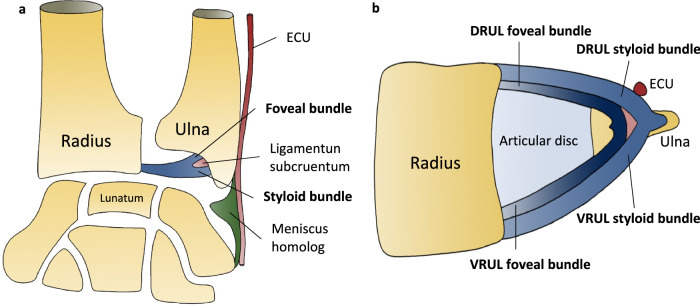
Illustrated anatomy of the triangular fibrocartilaginous complex: **a** coronal view of the DRUJ showing the styloid and foveal bundles. **b** Axial view from below illustrating the dorsal and volar radioulnar ligaments, each composed of two bundles, converging on the ulnar side to form styloid and foveal attachments. VRUL, volar radioulnar ligament; DRUL, dorsal radioulnar ligament; ECU, extensor carpi ulnaris

The triangular fibrocartilaginous complex is the primary component of the distal radioulnar joint (DRUJ) and serves as a key stabilising structure of the wrist [3, 4]. It is composed of seven elements: the articular disc, the dorsal and volar radioulnar ligaments (DRUL, ventral radioulnar ligament [VRUL]), the ulnolunate and ulnotriquetral ligaments, the deep portion of the extensor carpi ulnaris (ECU) tendon sheath, and the meniscus homologue [5]. The two main stabilising elements are the dorsal and volar radioulnar ligaments, which form a ring around the articular disc and insert on both the radius and the ulna [6]. Their ulnar insertion is dual, consisting of a styloid bundle and a foveal bundle, separated by a vascularised connective tissue known as the ligamentum subcruentum [7] (Fig. 1).

These two bundles may be injured either independently or simultaneously, and their assessment using arthro-CT remains a diagnostic challenge for radiologists. Moreover, traumatic avulsion of the radioulnar ligaments from the ulnar fovea is a recognised cause of DRUJ instability and has a greater impact on joint stability than lesions involving other insertional structures [8].

### Surgery

Surgical reattachment of the avulsed radioulnar ligaments to the foveal insertion of the TFCC is now recognised as the most effective approach for the management of TFCC tears [9]. Recent surgical techniques, such as arthroscopic ligament-specific repair of TFCC foveal tears, have been shown to reliably restore DRUJ stability, improve wrist range of motion, and enhance patient function [10].

However, isolated foveal lesions are not typically visualised during conventional radiocarpal arthroscopy, where the integrity of this attachment is assessed indirectly via the trampoline test or hook test [11]. The radiologist plays a key role in identifying these foveal lesions, which may guide surgeons toward a radioulnar arthroscopic approach, allowing for direct display of the foveal insertion [12].

### TFCC classification

Several classifications have been proposed in recent years to describe TFCC lesions, such as the Atzei [13], Zhan [14], and CUP [15] classifications, aiming to complement Palmer’s initial system [16], which does not account for isolated foveal lesions. Each classification has its own advantages and limitations, and its use largely depends on the radiologist’s experience.

### Objective

The primary aim of this study was to demonstrate that foveal lesions of the TFCC are underreported on arthro-CT. The secondary objective was to compare imaging review data with clinical and surgical findings to assess their impact on patient management.

## Materials and methods

This retrospective, observational, single-centre study was conducted in a specialised musculoskeletal imaging department.

All procedures performed in studies involving human participants were in accordance with the ethical standards of the institutional and national research committees and with the 1964 Helsinki declaration and its later amendments or comparable ethical standards. Approval was obtained from the French Ethics Committee for Research in Imaging (CERF), under reference number CRM-2507-491. The participants were informed of the study and given the opportunity to opt out, in accordance with ethical standards.

### Inclusion and exclusion criteria

Data were collected from patients who underwent wrist arthro-CT between January 2021 and December 2023 over a three-year period.

The exclusion criteria included moderate to severe wrist degenerative changes (grade 3 or higher according to the Kellgren and Lawrence classification [17]), a history of prior wrist surgery, absence of DRUJ opacification, or imaging artefacts precluding confident interpretation.

### Data collection

Arthrography was performed via a Philips MultiDiagnost Eleva system (Philips Medical Systems Nederland B.V.), following injection and opacification of the midcarpal, radiocarpal, and radioulnar compartments. Wrist arthro-CT was performed via a 64-slice GE Discovery 750HD scanner (GE Healthcare), with acquisitions at 100 kV and 150 mAs, generating 0.625 mm slice thickness images. Reconstructions were performed in soft tissue and bone windows in the coronal and sagittal planes.

**Fig. 2:**
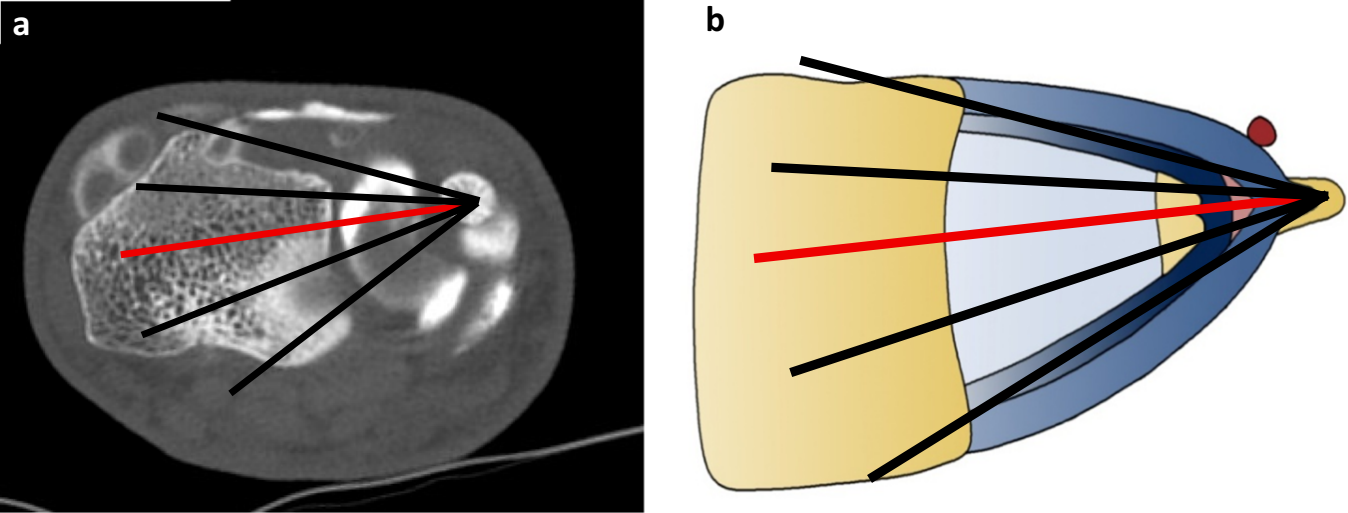
MPR. **a**, **b** Red line: standard coronal reconstruction. Black lines: fan-shaped coronal reconstructions centred on the ulnar fovea

The radiological data were reviewed by two radiologists in consensus, who were blinded to the initial reports and clinical information. In cases of discrepancy, a consensus reading was performed, with arbitration by a senior musculoskeletal radiologist with experience in wrist imaging. Interobserver agreement was not formally assessed. Both radiologists had undergone prior training in updated TFCC classifications and had experience with 20 confirmed cases from another database between January 2024 and December 2024. TFCC assessment was performed via multiplanar reconstructions (MPRs) centred on the styloid and fovea, as recommended by Jan-Peter Grunz et al [18]. This technique consists of fan-shaped coronal reconstruction of the TFCC centred on the ulnar fovea, allowing optimal display of both the dorsal and the volar ulnar attachments of the TFCC (Fig. 2).

TFCC lesions were classified according to their different anatomical components:

- Central portion: 1. normal (no perforation, regular thickness), 2. traumatic lesion (eccentric location, sharp and well-defined margins, cleft-like appearance, horizontal fissure, or bucket-handle tear), 3. degenerative lesion (central location, poorly defined margins, oval shape, or diffuse thinning).
- Radial portion: 1. Normal, 2. Perforated.
- Ulnar portion: 1. Normal, 2. Foveal bundle tear (detailed in complete lesion, partial dorsal or partial volar), 3. Styloid bundle tear, 4. Complete ulnar tear (involving both foveal and styloid bundles).
- Peripheral structures, including the ulnocarpal ligaments (1. normal, 2. tear) and the deep sheath of the ECU tendon (1. no sheath opacification, 2. sheath opacification).

Foveal lesions were defined using predefined imaging criteria. A lesion was considered present when contrast extension reached the foveal footprint, corresponding to the flat portion of the ulnar foveal surface [3], and when the abnormality was consistently identified on at least two orthogonal planes, typically coronal and sagittal reconstructions. Features suggestive of true tears included irregularity or disruption of ligament fibres and extension towards the radioulnar ligaments. In contrast, anatomical variants of insertion were characterised by smooth, well-defined margins and a homogeneous appearance, without clear ligamentous disruption [19].

TFCC data were also extracted from the initial radiology reports, which were produced by radiologists specialising in musculoskeletal imaging as part of their routine clinical practice. For each patient, we recorded whether a TFCC lesion was reported, as well as the type and location of the lesion, as previously described. These findings were then compared with the re-evaluation data.

Clinical data were extracted from reports of orthopaedic consultations conducted by hand and wrist specialists. These data included a history of trauma, the time interval between trauma and arthro-CT, presence and location of pain, and instability assessed by the radioulnar ballottement test [20].

Additionally, we assessed whether patients had undergone surgery at our institution. Operative reports were reviewed to identify TFCC-related keywords and evaluate concordance between surgical findings and imaging review data.

### Statistical analysis

Descriptive statistics were used for demographic data. The results are presented as the means ± standard deviations (SDs) and ranges or medians ± interquartile ranges (IQRs, Q1–Q3) for continuous variables, and as counts and percentages for categorical variables.

For the primary outcome measure, statistical analyses were conducted to compare the findings from the imaging review with the initial radiological reports to determine the extent of underreporting of TFCC lesions.

Additional statistical analyses were performed to explore potential associations between clinical findings—particularly pain and instability—and lesion types as part of a hypothesis-generating approach regarding their potential impact on patient management. Data analysis was performed via GraphPad Prism version 8.00 (GraphPad Software, LLC). Comparisons between independent groups were conducted via Fisher’s exact test, with statistical significance set at *p* < 0.05.

Given the limited number of complete cases and the proportion of missing clinical data, multivariate analysis was not performed because of the risk of overfitting and unreliable estimates.

## Results

**Fig. 3:**
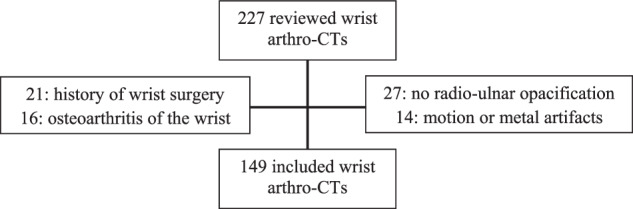
Flow chart detailing the number of patients excluded on the basis of the technical and imaging criteria

Among the 227 reviewed wrist arthro-CTs, 149 were included in the study, and 78 were excluded on the basis of predefined exclusion criteria (Fig. 3).

### Descriptive population analysis

The study population comprised 100 men (67%) and 49 women (33%), with a mean age of 33.5 ± 12 years, ranging from 15–70 years. A history of trauma was reported in 106 patients (71%), whereas trauma was reported in 23 patients (16%). The mean interval between trauma and arthro-CT was 6.6 months, with a median of 2 months (interquartile range: 1–6 months). Pain was reported in 138 patients (92.6%), including ulnar-sided pain in 50 patients (34%). Radioulnar instability was present in 14 patients (9.4%), absent in 54 patients (36.2%), and undocumented in 81 patients (54.4%) (Table 1).

**Table 1:** Population characteristics and clinical data

Total patients: *n* (%)	149 (100)
Sex: *n* (%)	
Men	100 (67)
Women	49 (33)
Age (years): mean ± SD [min-max]	33.5 ± 12 [15–70]
Trauma: *n* (%)	
Yes	106 (71)
No	23 (16)
ND	20 (13)
Trauma delay (months):	
Median [Q1–Q3]	2 [1–6]
Mean [min–max]	6.6 [0.25–72]
Pain and location: *n* (%)	
Ulnar	50 (34)
Medio-carpal	30 (20)
Dorsal	10 (7)
Radial	9 (6)
Palmar	3 (2)
ND	42 (28)
No pain	5 (3)
Radio-ulnar instability: *n* (%)	
Yes	14 (9.4)
No	54 (36.2)
ND	81 (54.4)

*ND* no data, *SD* standard deviation

### Descriptive lesion analysis

On imaging review, TFCC lesions were identified in 88 patients (59%), whereas no lesions were identified in 61 patients (41%) (Table 2). The distribution of these lesions was as follows: 49 (33%) central, 64 (43%) ulnar, and 5 (3%) radial. Central lesions included 40 (27%) traumatic lesions and 9 (6%) degenerative lesions. The ulnar lesions included 42 (28%) isolated foveal lesions, 8 (6%) styloid lesions, and 14 (9%) complete ulnar lesions. Among the foveal lesions, 20 (13%) were complete tears, 19 (13%) involved the dorsal portion of the foveal bundle, and 3 (2%) involved the volar portion.

**Table 2:** Distribution of TFCC tear

Distribution of TFCC tear	Initial report: *n* (%)	Image review: *n* (%)
Total patients	149 (100)	149 (100)
TFCC tear		
Yes	71 (48)	88 (59)
No	78 (42)	61 (41)
Central portion of the TFCC		
Normal	**104 (70)**	**100 (67)**
Tear	**45 (30)**	**49 (33)**
Traumatic lesion	38 (25)	40 (27)
Degenerative lesion	7 (5)	9 (6)
Ulnar portion of the TFCC		
Normal	**125 (84)**	**85 (57)**
Tear	**24 (16)**	**64 (43)**
Complete ulnar tear	9 (6)	14 (9)
Foveal tear	11 (7)	42 (28)
Foveal complete	5 (3)	20 (13)
Partial dorsal	6 (4)	19 (13)
Partial volar	0	3 (2)
Styloid tear	4 (3)	8 (6)
Radial portion of TFCC		
Normal	**145 (97)**	**144 (97)**
Tear	**4 (3)**	**5 (3)**

Bold values refers to the main categories

With respect to the peripheral components of the TFCC, ECU sheath lesions were identified in 10 patients (7%). No ulnocarpal ligament involvement was observed.

**Fig. 4:**
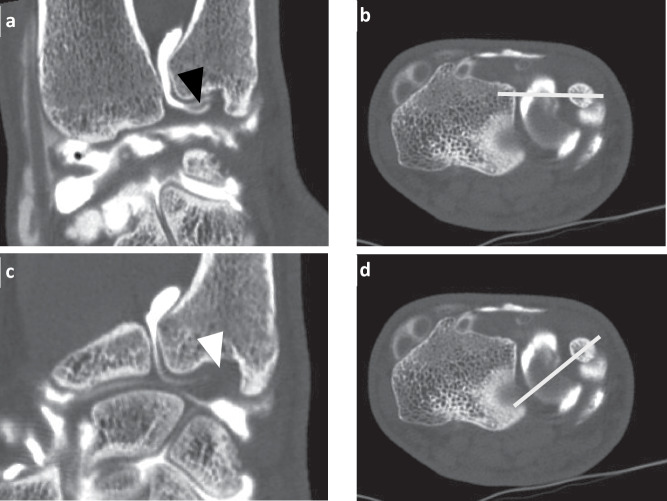
Arthro-CT of a 31-year-old woman with ulnar-sided wrist pain and no radioulnar instability; no lesions were described in the initial report. The review shows a partial foveal tear with preservation of the styloid bundle. **a**, **b** Dorsal foveal fissure visible on a dorsal radial plane (black arrow), corresponding to the white line on axial view. **c**, **d** Intact appearance of the volar portion of the foveal attachment (white arrow) on a volar radial plane

A total of 45 discrepancies were identified when the results of the imaging review were compared with those of the initial reports. Of these, 40 (89%) involved ulnar-sided lesions, including 31 (69%) foveal tears (Fig. 4), 4 (9%) styloid bundle tears, and 5 (11%) complete ulnar tears. Only one discrepancy (2%) involved a radial lesion (Table 3). Additionally, 4 discrepancies (9%) concerned central lesions, all of which were associated with concomitant discrepancies in the ulnar attachment.

**Table 3:** Discrepancy between the initial report and proofreading *n* (%)

Total patients	149 (100)
Discrepancy characteristics	**45 (100)**
Central tear	**4 (9)**
Ulnar tear	**40 (89)**
Complete ulnar tear	5 (11)
Foveal tear	31 (69)
Styloid tear	4 (9)
Radial tear	**1 (2)**

Bold values refers to the main categories

### Clinical analysis

Patients with complete ulnar TFCC lesions reported significantly more ulnar-sided pain (12 patients, 85.7%) than those without TFCC lesions (10 patients, 16.4%) (*p* < 0.0001). Similarly, radioulnar instability was significantly more common in the complete ulnar lesion group (7 patients, 35%) than in the group without TFCC lesions (3 patients, 4.9%) (*p* = 0.0019).

Among patients with foveal lesions, those with ulnar-sided pain were significantly more common (16 patients, 38.1%) than were those without TFCC lesions (10 patients, 16.4%) (*p* = 0.02). However, there was no significant difference in the prevalence of radioulnar instability between the foveal lesion group (3 patients, 7.1%) and the group without TFCC lesions (3 patients, 4.9%) (*p* > 0.99).

### Surgical data

Among the 149 patients, 12 underwent TFCC surgery, 8 underwent TFCC surgery alone, 3 underwent TFCC surgery combined with scapholunate surgery, and one underwent TFCC surgery combined with dorsal capsulo-scapholunate septum (DCSS) surgery. Among these 12 patients, 8 underwent surgery on the basis of TFCC lesions identified in both the initial radiology reports and the review, showing complete agreement (6 complete ulnar tears, 1 styloid tear, and 1 traumatic central lesion). The remaining 4 underwent surgery despite the absence of TFCC abnormalities in the initial reports. However, the review confirmed the presence of TFCC lesions in all 4 patients (2 styloid tears, 2 complete ulnar tears).

Among the 31 patients with foveal attachment discrepancy, 29 patients reported wrist pain (11 on the ulnar side). Only one case of radioulnar instability was observed, and no surgery was performed. For the 4 styloid attachment discrepancies, all patients reported pain (1 with instability), and 2 underwent surgery. Among the 5 patients with complete ulnar attachment discrepancy, 4 patients reported wrist pain (2 with instability), and 2 received surgical treatment.

## Discussion

In this retrospective study, we analysed 149 wrist arthro-CTs performed between 2021 and 2023. Our aim was to conduct a secondary review using the most recent knowledge and updated classification systems.

This reassessment revealed underreported lesions in 42 patients, representing 28% of the scans analysed. Most of these lesions involved the ulnar attachment of the TFCC, with 40 cases (89%) not reported on initial readings. These included 31 foveal lesions (69%), 4 styloid lesions (9%), and 5 complete ulnar lesions (11%). In addition, 4 underreported central lesions and 1 radial lesion were identified.

The predominance of undetected foveal attachment lesions in our study is consistent with previous reports emphasising the diagnostic challenge for this type of injury. Bille et al [21] reported a low sensitivity (30%) but high specificity (94%) for detecting foveal tears via arthro-CT. Similarly, Welling and Kakar [22] highlighted the poor sensitivity of MRI, with only 8 foveal tears identified among 23 arthroscopically confirmed cases.

**Fig. 5:**
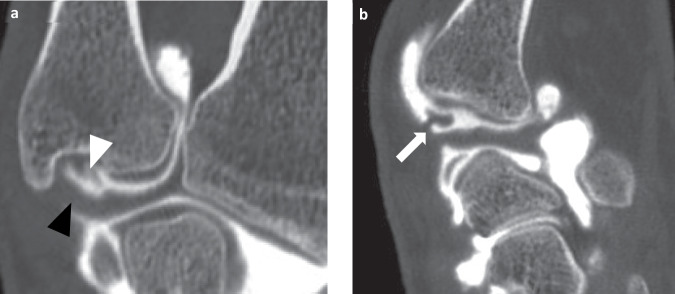
Arthro-CT of a 33-year-old man with ulnar-sided wrist pain and no radioulnar instability; no lesions were described in the initial report. **a** Coronal view of the TFCC showing an isolated tear of the foveal bundle (white arrowhead) with an intact styloid bundle (black arrowhead). **b** Sagittal view demonstrating dorsal extension of the tear into the DRUL (white arrow)

Among the foveal lesions, 86% involved the dorsal part of the attachment (Fig. 5), corresponding to a tear in the dorsal bundle of the distal radioulnar ligament. This ligament is an important stabiliser of the DRUJ [23] and is therefore a potential contributor to instability [24].

Several factors may explain the underreporting of foveal lesions. First, this may be due to a lack of awareness of this type of injury. Additionally, partial involvement of the ulnar attachment does not result in communication between the radioulnar and ulnocarpal compartments, unlike complete ulnar tears, which are more frequently reported. Finally, even among radiologists familiar with these injuries, foveal bundle involvement is often partial and most frequently affects the dorsal aspect [23]. These lesions must be assessed along the entire foveal attachment, from dorsal to palmar aspects, using MPR centred on the ulnar styloid and sagittal views rather than limiting the analysis to the mid-fovea [18].

Current classification systems have their own strengths and limitations. Although simple classifications such as Palmer’s classification are easy to use, they do not address foveal lesions. More comprehensive systems (Atzei, Zhan, and CUP) are often too complex for routine practice and still overlook certain variants. In daily use, their application depends on local habits; therefore, we advocate a standardised anatomical descriptive approach in collaboration with surgeons.

In terms of clinical outcomes, patients with TFCC lesions showed a higher prevalence of ulnar-sided pain. This association remained significant for both foveal and total ulnar lesions, in line with previous studies suggesting a role of foveal lesions in DRUJ pain. However, these findings should be interpreted with caution, as a substantial proportion of patients had missing clinical data, particularly regarding pain localisation and DRUJ instability, which may have introduced bias and affected the strength of the observed associations [10–12]. No significant difference in DRUJ instability was observed in patients with isolated foveal lesions. Welling et al [22] reported that the absence of clinical instability does not rule out the presence of a foveal lesion. These results may be explained by the partial nature of these lesions, which may not cause instability. Conversely, complete ulnar lesions (involving both foveal and styloid attachments) were associated with a higher prevalence of DRUJ instability. However, given the limitations related to missing data, this association should be interpreted cautiously, although it remains consistent with biomechanical studies highlighting the critical role of these attachments in joint stability [6, 8, 25, 26].

Among the 149 patients, only 12 underwent TFCC surgery. Eight of these lesions were identified in the initial report, with intraoperative findings confirming the diagnosis. However, 4 patients underwent surgery despite the absence of abnormalities on their initial imaging studies. In these cases, retrospective imaging review confirmed the lesions, with findings consistent between the review and surgical reports.

Diagnostic wrist arthroscopy remains difficult. Andrew Park et al [27] reported significant interobserver and intraobserver variability in the diagnosis of TFCC lesions by arthroscopy, with an interobserver kappa coefficient of 0.33 and an intraobserver coefficient of 0.88. The variability may be due to the standard surgical approach, which accesses the TFCC from the carpal side, limiting direct display of radioulnar lesions [28]. Furthermore, partial lesions without associated instability may yield negative results on diagnostic tests performed intraoperatively. This highlights the importance of accurately describing foveal lesions, which are also challenging to diagnose arthroscopically. Identifying such a lesion on imaging may prompt the surgeon to adopt a direct radioulnar approach.

The strength of our study lies in its integration of the latest TFCC lesion findings and classification systems. Reviewing a large series of arthro-CT, combined MPR techniques [18], has improved the sensitivity of lesion detection, particularly for foveal injuries. Therefore, the improved accuracy provided by the radial plane view for the detection of ulnar-sided TFCC lesions is highly clinically relevant.

These results could also be extrapolated to MRI. However, the absence of intra-articular opacification in standard MRI may limit the detection of such lesions, which are primarily identified through the presence of contrast at the foveal footprint level [29]. MR arthrography appears to be a good compromise, although its lower spatial resolution may also constitute a limitation for identifying the most subtle lesions. The use of the MPR on 3D MR arthrography sequences could help improve the detection of these lesions. However, further studies are needed to assess the accuracy of CT arthrography in diagnosing peripheral TFCC tears compared with that of MR arthrography.

However, this study has several limitations. The lack of patient follow-up prevents the assessment of clinical outcomes. Owing to the retrospective nature of the study, a substantial proportion of patients had missing clinical data, particularly regarding DRUJ instability and pain localisation. This may have introduced bias and reduced the statistical power of analyses, potentially affecting the strength of the observed associations, especially for instability. As previously mentioned, it is crucial to correlate imaging findings with clinical symptoms, given the possibility of asymptomatic TFCC lesions. Another limitation of this study relates to the imaging analysis methodology. The consensus approach ensured a consistent final interpretation; however, it prevents objective evaluation of reproducibility and may have led to an overestimation of diagnostic performance. A major limitation of this study is the absence of surgical confirmation for foveal lesions, particularly partial foveal lesions, none of which were surgically validated. The use of standardised imaging criteria may help reduce the risk of misclassification between true lesions and anatomical variants, although this limitation cannot be completely eliminated in the absence of systematic surgical correlation.

Nevertheless, the objective of this study was to raise awareness of the existence of these lesions to reduce the risk of false negatives and to support surgical decision-making when a foveal lesion is identified in accordance with clinical findings.

In conclusion, the application of newly published classifications and radial sequences improved the diagnostic accuracy for peripheral lesions, enabling us to identify previously unreported lesions in 28% of cases. This study revealed that foveal lesions are frequently underreported, leading to chronic ulnar pain that is disabling for sports and professional activity in young and active people. In combination with clinical examination, communication with the orthopaedic surgeon is crucial to ensure optimal patient management.

SYSTEMATIC CHECKLIST FOR TFCC ASSESSMENT ON WRIST ARTHRO-CT

1. On a strict coronal plane, perform an initial overall assessment of the radial, central, and ulnar portions of the TFCC.
2. MPRs centred on the ulnar styloid should be performed to assess the dorsal and volar components of the ulnar TFCC attachment, including both the foveal and styloid insertions. Particular attention should be paid to the foveal attachment, with identification of the foveal footprint (the flat surface at the base of the fovea) and assessment for deep contrast extension to this region.
3. Confirm the lesion on a sagittal plane, with particular attention to the extension of a fissure toward the dorsal or volar radioulnar ligaments.
4. The pathological nature of the fissure should be confirmed by assessing its morphology, with particular attention to irregular margins, ligament fibre disruption, and poorly defined contours, as opposed to the smooth and well-circumscribed appearance of anatomical variants.
5. Assess the peripheral structures, including the dorsal and volar capsules, the deep aspect of the ECU tendon sheath, and the ulnocarpal ligaments.

## Data Availability

Data sets generated during the current study are available from the corresponding author on reasonable request.

## Abbreviations

DRUJ: Distal radioulnar joint
DRUL: Dorsal radioulnar ligament
ECU: Extensor carpi ulnaris
MPR: Multiplanar reconstruction
TFCC: Triangular fibrocartilage complex
VRUL: Ventral radioulnar ligament

## Declarations

### Methodology

- Retrospective
- Observational
- Single institution
